# Individuals with covert severe acute respiratory syndrome coronavirus 2 infection: Are they a critical booby-trap?

**DOI:** 10.1590/0037-8682-0231-2020

**Published:** 2020-06-01

**Authors:** Fatma Abdelaziz Amer

**Affiliations:** 1 Zagazig Faculty of Medicine, Department of Medical Microbiology and Immunology, Zagazig, Egypt.

**Dear Editor:**

I believe that in Egypt, the first phase of the coronavirus disease (COVID-19) pandemic was characterized by symptomless (covert) carriers. Covert carriers are individuals who test positive for the virus on laboratory testing but are symptomless and can shed the virus. Numerous researchers assume that there is an unobserved pool of these carriers because in many cases, severe acute respiratory syndrome coronavirus 2 (SARS-CoV-2) infections could not be related to contact with persons with infection or to travel to epidemic areas. Recent studies have revealed that some populations may have up to 50% of covert carriers^1^. The sequence of events leading up to the current full-blown epidemic may provide some explanation on this aspect. On February 14, 2020, the first case of SARS-CoV-2 infection was confirmed in Egypt in a Chinese national^2^. He was diagnosed on arrival in Cairo. His lengthy stay in a confined place (the plane) provided a good opportunity for virus shedding (the time required to travel from China to Cairo is nearly 15 hours). Considering the additional time taken to arrive at the testing area, the person had sufficient time for transmitting the virus to the surroundings. At that time, SARS-CoV-2 testing of all contacts showed negative results. 

On February 28, four foreigners who had travelled to Egypt were reported to be infected: two French nationals, one Canadian national, and one Taiwanese national^3^. At that time, not a single case of SARS-CoV-2 infection had been recorded in Egypt, which raises many inquiries. The next incident which added to the dilemma was provided by the Egyptian Cruise Ship MS A’ Sara in late February and early March. Nearly 18 Americans were reported to have SARS-CoV-2 infection after their voyage to Luxor”? . Investigations revealed that 12 of the crew members were positive for SARS-CoV-2 and that the index case involved a Taiwanese-American female tourist^4^, who had traveled on the ship between late January and early February. She apparently passed on the virus to the crewmembers. None of the 12 crew members positive for SARS-CoV-2 showed any symptoms. At that time, no cases of SARS-CoV-2 infection had been officially reported by the Egyptian Health Authorities. 

On March 6, three overt cases involving Egyptian nationals were recorded. By March 9, 59 COVID-19 cases had been announced^4^. This indicates that with the initiation of exposure on February 14^2^, nearly 1 month passed until the appearance of obvious cases; this appears to be a long duration in a country of 100 million people. Furthermore, all patients who tested positive for SARS-CoV-2 earlier^3^ had neither signs nor symptoms. A convincing explanation for this epidemiological puzzle is that the SARS-CoV-2 caused covert/asymptomatic infection in patients over this period. As reported by researchers from the United States of America^1^, asymptomatic patients play a major role in the transmission of SARS-CoV-2.

**FIGURE 1:**
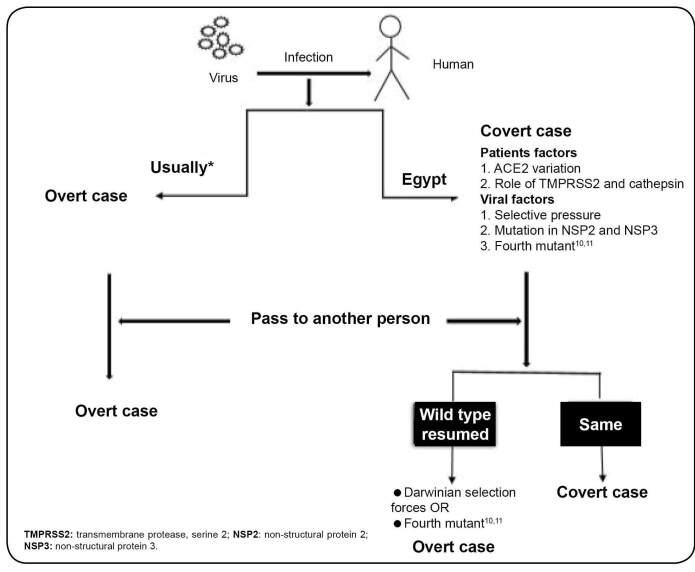
Schematic representation of the postulated sequence involved in the severe acute respiratory syndrome coronavirus 2 infection outbreak over time among Egyptians. Host and/or viral factors may affect patient/virus interaction. Host factors include ACE2 receptor, and other enzymes priming the entry process, e. g., TMPRSS2 and cathepsin. Viral factors include mutation or other subtypes. When the virus infects others, it may remain covert. Overt infection usually results owing to potency restoration (wild type) due to Darwinian selection force or the emergence of a fourth mutant. *Usually: most of the cases but not all.

After March 6, overt and symptomatic cases have continued to appear. Postulated sequences involved in the SARS-CoV-2 outbreak over time among Egyptians are shown in Figure 1. By May 6, a total of 7201 cases have been confirmed. A thorough analysis of data carried out on a scientific basis and guided by the most recent publications resulted in the following important preliminary conclusions. In addition to research on the important role of covert carriers in the initiation of the pandemic, more research is needed on the type of patient-virus interactions encountered among Egyptians. Theoretically, this interaction can be attributed to host and/or viral factors. Host factors are related to angiotensin converting enzyme 2 (ACE2). ACE2 is known as the key human target for the attachment of the viral spike (S) protein, which is followed by entry of SARS-CoV-2 into cells with subsequent viral multiplication^5^. Since the expression level and pattern of ACE2 vary^6^ ^,^ ^7^, various forms and degrees of disease may be expected. 

Viral factors that may be implicated in patient-virus interactions include a possible change in viral characteristics due to selective pressure, which may be either biotic or abiotic^8^. Another significant factor is virus mutation^9^. Very recently, it has been assumed that there are three distinct mutants of SARS-CoV-2^10^, which raises the possibility of other mutants in other geographic locations with differences in the type of clinical infections and outcomes. Very recently, researchers from England^11^ discovered at least 12 strains of coronavirus in the United Kingdom (UK), one of which has only ever been found in the UK; this indicates that this virus mutated on British soil. 

The magnitude of asymptomatic individuals with SARS-CoV-2 infection or other infections represent a critical medical problem. A recent article has shown an increase in the proportion of asymptomatic carriers of severe acute respiratory syndrome coronavirus (SARS-CoV) from 0% to 28.6%^12^. 

Contrary to the generally acknowledged notion that early on in the course of any outbreak, the detection of severe cases is followed by that of less severe cases (paucisymptomatic or asymptomatic), it appears that SARS-CoV-2 transmission started in Egypt with asymptomatic carriers. Perhaps other outbreaks worldwide also started in a similar manner; it is possible that people were not ill enough to seek medical help or to undergo appropriate testing. However, after the infection of more than 3 million people and the continuous spread of COVID-19 worldwide, a new approach is urgently needed that expands COVID-19 testing to include asymptomatic persons in prioritized settings^1^.

Further investigation is required to determine whether all of the above assumptions are true or false and whether they are applicable to various geographic locations and different ethnic groups. Indeed, it is crucial to understand the natural history of coronavirus outbreaks in different geographic locations so as to allow policy makers to plan sound prevention strategies, adjust the length bias for the effective implementation of surveillance programs, and to predict prognosis and evaluate interventions. Moreover, intensive research at a molecular level is required to characterize the subtypes of SARS-CoV-2 prevalent in Egypt.
